# Impact of Co-Designed Game Learning on Cultural Safety in Colombian Medical Education: Protocol for a Randomized Controlled Trial

**DOI:** 10.2196/17297

**Published:** 2020-08-31

**Authors:** Juan Pimentel, Anne Cockcroft, Neil Andersson

**Affiliations:** 1 CIET-PRAM Department of Family Medicine McGill University Montreal, QC Canada; 2 Facultad de Medicina Universidad de La Sabana Chia Colombia; 3 Escuela de Medicina y Ciencias de la Salud Universidad del Rosario Bogota Colombia; 4 Centro de Investigación de Enfermedades Tropicales (CIET) Universidad Autónoma de Guerrero Acapulco Mexico

**Keywords:** transformative learning, medical education, cultural safety, participatory research, game jam

## Abstract

**Background:**

Cultural safety encourages practitioners to examine how their own culture shapes their clinical practice and to respect their patients’ worldviews. Lack of cultural safety in health care is linked to stigma and discrimination toward culturally diverse patients. Training in cultural safety poses considerable challenges. It is an unappealing subject for medical students and requires behavioral changes in their clinical practice. Game jams—collaborative workshops to create and play games—have recently shown effectiveness and engaging potential in university-level education.

**Objective:**

The trial aims to determine if medical students’ participation in a game jam to design an educational game on cultural safety is more effective than a standard lesson on cultural safety in terms of change in the students’ self-reported intended patient-oriented behavior.

**Methods:**

A parallel-group, 2-arm randomized controlled trial with a 1:1 allocation ratio will randomize 340 medical students and 60 medical interns (n=400) at the Faculty of Medicine at La Sabana University, Colombia (170 students and 30 medical interns to each arm). The intervention group will participate in an 8-hour game jam comprising (1) a preliminary lecture on cultural safety and game design, (2) a game building session where groups of students will create educational games about cultural safety, and (3) a play-test session in which students will play and learn from each other’s games. The control group will receive a standard lesson, including a 2-hour lecture on cultural safety, followed by a 6-hour workshop to create posters about cultural safety. Web-based self-administered 30-item Likert-type questionnaires will assess cultural safety self-reported intended behavior before, immediately after, and 6 months after the intervention. An intention-to-treat approach will use a *t*-test with 95% CIs to determine the significance of the effect of the intervention, including within- and between-group comparisons. The qualitative most significant change technique will explore the impact of the intervention on the clinical experience of the students.

**Results:**

Study enrollment began in July 2019. A total of 531 students completed the baseline survey and were randomized. Data collection is expected to be complete by July 2020, and results are expected in October 2020. The study was approved by the institutional review board of the Faculty of Medicine at McGill University (May 31, 2017) and by the Subcommittee for Research of the Faculty of Medicine at La Sabana University (approval number 445).

**Conclusions:**

The research will develop participatory methods in game-based learning co-design that might be relevant to other subjects. Ultimately, it should foster improved cultural safety skills for medical students, improve the quality of health services for diverse cultural groups, and contribute to enhanced population health. Game learning may provide an innovative solution to a long-standing and neglected problem in medical education, helping to meet the educational expectations and needs of millennial medical students.

**Trial Registration:**

ISRCTN Registry ISRCTN14261595; http://www.controlled-trials.com/ISRCTN14261595

## Introduction

### Cultural Safety Training

Although cultural safety is an evolving term and lacks a formal definition [1], it is often described as a space “that is spiritually, socially, emotionally and physically safe for people; where there is no assault, challenge or denial of their identity, of who they are, and what they need” [2]. The concept originated in New Zealand to address the disconnect between the type of health care that indigenous Maori people were receiving and the culturally congruent care that they were advocating for [3].

Cultural safety has gradually gained attention because it offers a more comprehensive and respectful way to approach culture, in many settings replacing the current standard, which is cultural competence [4]. Cultural safety is distinct from cultural competence, in that it invites culturally diverse patients and their communities to co-design and evaluate culturally safe health care [1,5]. The notion of participation in health care design also differentiates cultural safety from cultural humility [6], another well-known approach to cultural diversity in health care.

The Royal College of Physicians and Surgeons of Canada will soon require all medical residency programs to provide mandatory cultural safety training [7,8]. There is, however, little research on how to implement this approach in medical education [9], and how health professionals acquire cultural safety skills is poorly understood [10].

There are additional challenges to promoting cultural safety in medical education. Educators might find cultural safety complicated to teach, and medical students might perceive it as dull or, given the altruistic tone of their chosen profession, unnecessary for them [11]. Contemporary medical training is overloaded almost everywhere, with little space to include an entirely new if very important subject. Millennial medical students—the birth cohort between 1979 and 2000 [12]—have new learning relationships with technology, creativity, and amusement that modern teaching strategies cannot overlook [13]. Finally, cultural safety training goes beyond mere knowledge acquisition; it must promote a *transformative* experience to impact students’ behavior in clinical practice. The theory of transformative learning provides a framework to address these challenges [14].

### Transformative Learning and Game Co-Design

Mezirow describes transformative learning as a process that changes frames of reference, “the structures of assumptions through which we understand our experiences” [14]. Frames of reference comprise habits of mind, which are habitual ways of thinking and acting, and points of view, which are beliefs, values, and attitudes.

Mezirow argues that ethnocentrism, defined as “the predisposition to regard others outside one’s own group as inferior” [14], is an example of a habit of mind. Ramsden, the Maori nurse who developed the concept of cultural safety, proposes that confronting ethnocentrism must be the first step in cultural safety training [3]. Transformative learning may, therefore, be suitable for providing cultural safety training to medical students.

Transforming frames of reference requires reflection on the assumptions upon which learners base their habits of mind and points of view [14]. In transformative learning, people become critically reflective of their assumptions through education that is participatory and interactive and through group problem solving or communicative learning [15].

*Game jams* provide an environment to foster learning through interacting and communicating with others [16], an essential aspect of transformative learning. These participatory events allow attendees to create games (digital or board games) in a time-constrained environment [17]. Unlike other educational approaches, game jams could offer a solution to the challenges of cultural safety in medical education by (1) engaging millennial students through a culture of creativity and learning, play testing, and idea sharing; (2) supporting a transformative process of learning-by-doing while enhancing creative thinking, problem solving, communication, and innovation; and (3) promoting transformative learning in less time, thus offering an alternative to overloaded medical curricula.

Fowler et al [16] recently found that game jam participation could improve the performance of computing students. However, we are not aware of any reported experience using game jams to train medical students. Our primary objective is to determine if medical students’ participation in a game jam to design an educational game on cultural safety is more effective than a standard lesson on cultural safety in terms of change in students’ self-reported intended patient-oriented behavior. Our secondary objectives are to (1) determine the impact of the intervention (game jam) compared with the control (standard lesson on cultural safety) on students’ confidence in their general transcultural skills and (2) assess the impact of participation in the game jam through a narrative approach that identifies in their own words the effect of the learning on cultural safety in their clinical practice.

## Methods

### Trial Design

A parallel-group, 2-arm, randomized controlled trial (RCT) with 1:1 allocation will compare participation in a game jam with a standard lesson on cultural safety. The RCT will answer the following question:

- Among medical students and interns from La Sabana University, does participating in a game jam for cultural safety training, in comparison with a standard lesson on cultural safety, result in an increased change in students' and interns’ (1) self-reported intended behavior, (2) confidence in general transcultural skills, and (3) reported change in clinical practice?

Textbox 1 presents the population, intervention, contrast, outcomes, and time points components of the research question. This protocol description follows the standard protocol items: recommendations for interventional trials 2013 statement [18] (Multimedia Appendix 1).

**Population, intervention, contrast, outcome, and timing of the randomized controlled trial.**

Population

- Undergraduate medical students and medical interns at La Sabana University in Colombia

Intervention

- Game jam aimed at fostering cultural safety in clinical practice

Contrast

- Standard lecture and workshop on cultural safety

Outcome

- (1) Cultural safety–intended patient-oriented behavior change outcomes from knowledge to action, (2) students’ confidence in general transcultural skills, and (3) qualitative understanding of the change experienced by participants in their clinical practice

Timing

- Before the intervention, immediately following the teaching session, and 6 months after the intervention

### Study Setting

We will conduct the RCT at the Faculty of Medicine at La Sabana University in the municipality of Chía, Colombia. Chía is a small town located 15 km from Bogotá, the capital of Colombia. La Sabana University is a private higher education institution that has 8926 undergraduate students; 22% of these students come from a low socioeconomic level, 52% belong to the middle class, and the remaining 26% come from higher socioeconomic backgrounds [19]. Presently, there are 956 students enrolled in the medical school and 256 medical interns (n=1212) [19]. At La Sabana, the duration of the doctor of medicine program is 7 years. As part of this training, all medical students must undergo a one-and-a-half-year medical internship before graduating.

### Eligibility Criteria

The inclusion criteria are as follows: (1) being a medical student or medical intern at any level of training and (2) providing informed consent. The exclusion criterion is not wanting to participate in the study.

### Interventions

#### Game Jam

**Figure 1:**
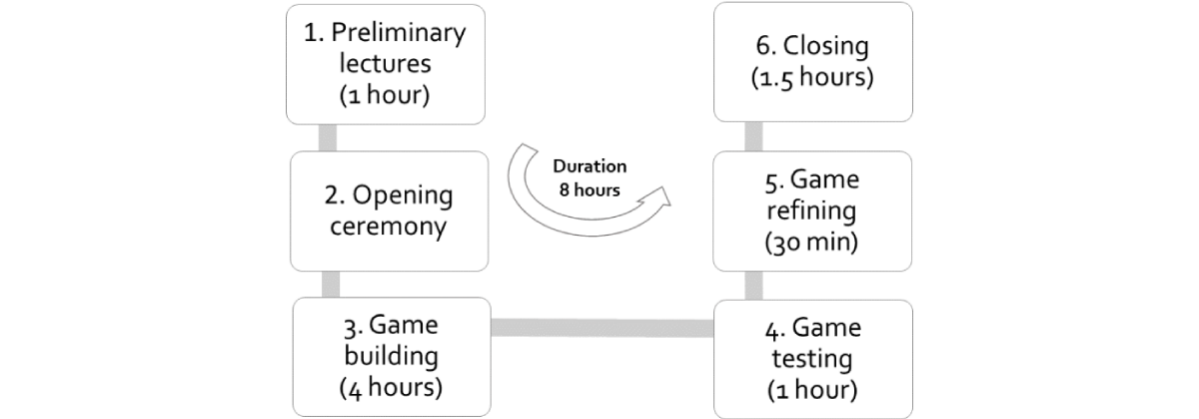
Game jam protocol.

The intervention will consist of a game jam aimed at creating a low-technology prototype of an educational game to foster cultural safety in medical education. Groups of 5 or 6 students or medical interns will create an educational game prototype from scratch. We will follow the 6-step game jam protocol based on Macklin’s *planning your game jam* guidelines [20] (Figure 1):

(1) Preliminary lecture session (1 hour): this comprises a 30 min lecture on cultural safety, based on a cultural safety curriculum co-designed with local community members knowledgeable about cultural and traditional health practices [21], and a 30 min lecture on game design.

(2) Opening ceremony: game jams usually start with opening comments from the host. We will welcome the participants and share the agenda and rules of the game jam.

(3) Game building (4 hours): this includes 6 steps:

- We will invite participants to write a brief narrative of when they witnessed (or heard of) discrimination or disrespect against a patient because of their traditional health practices and the consequences of this discrimination.
- Participants will share their brief stories within their game jam group to discuss and select the story (based on consensus) that best describes discrimination or disrespect against a patient. A key component of this step is to imagine and brainstorm the fullest range of possible consequences—from trivial to life-threatening.
- Participants will anonymize the selected story as that of a fictional medical student who has to undergo a primary care clinical rotation in a local community where she or he faces intercultural tensions in clinical practice. The participants will then convert this narrative into a game and define a set of rules, rewards, and penalties.
- Participants will discuss the factors that hypothetically lead the medical student to be discriminatory or disrespectful toward his or her patient in the story. After the discussion, each group will select and integrate 5 to 10 factors into the game. The challenge here is that players have to become aware gradually that these factors can lead to disrespect or discrimination against culturally diverse patients as they play the game. Concretely, the jammers will be expected to add factors such as the hegemony of evidence-based medicine, colonization and ethnocentrism, and other factors defined in the co-designed cultural safety curriculum.
- Participants will discuss what can be done to address each of the selected factors that contributed to the disrespect or discrimination experienced by the patient in the narrative. Each group will select and integrate 5 to 10 actions to promote dialogue and respect toward culturally diverse patients in clinical encounters into their game. The *challenge* is that players learn to respect patients who use traditional health practices in clinical encounters as they play the game.
- The students will discuss and identify ideas to start working with the patient as a team in the health care decision-making process. This involves engaging in dialogue with the patient to invite them to bring their cultural and traditional practices to inform the health care decision-making process. Traditional practices will be predefined by our co-designed curriculum [21]. The *challenge* is that players of the game have to learn how to work with traditional medicine users to make health decisions that are culturally safe jointly.

(4) Game testing (1 hour): groups will learn from each other’s solutions, ideas, and resources, thus strengthening the cultural safety learning process. At least one member of each group will stay at their workplace to present their game. The remaining students of the group will rotate to play the games created by other groups, thus ensuring that participants from all groups will play at least two additional games. Before the end of the session and using Google Forms (Google LCC), we will ask the students to evaluate other groups’ games in different categories aligned with each of the challenges.

(5) Game refining (30 min): after playing and testing other teams’ games, each group will have new ideas for refining their own game. Groups will then return to their workplace and apply lessons to improve their own game. Each group will fill a form to register their game on Google Forms.

(6) Closing (1 hour 30 min): we will bring the full group together for the final presentation of the games. Each group will have to provide a brief description of their game and discuss how they solved each of the game building challenges. We will facilitate this session to highlight the underlying concepts of cultural safety. Finally, we will award prizes in 3 different categories aligned with each of the challenges.

#### Control Group

The control group will receive a 1 hour 30 min lecture on cultural safety in medical education by an expert in cultural safety. The lecture will be a standard lesson using PowerPoint slides and will cover the same key concepts used in the game jam, including (1) definition of cultural safety, (2) consequences of cultural tensions in health care, (3) self-awareness, (4) Colombian cultural health practices, and (5) respect for culturally diverse patients. The lecture will be based on our co-designed curriculum [21]. The session will be followed by a 15-min period to make comments and to ask questions and a 15-min break.

After the break, the students will participate in a 6-hour workshop based on cultural safety selected readings. Groups of 5 or 6 students or medical interns will answer 10 open-ended questions based on the lecture and the readings. They will create a poster to graphically display their responses to other students. Similar to the game jam session, we will split each group and encourage a rotation process where participants from all groups will learn from at least two additional posters. Before the end of the session and using Google Forms, we will ask the students to evaluate the other groups’ posters in 4 different categories: creativity, coverage of the topic, graphics and pictures, and layout and design.

In the closing session, the best groups will present their posters to the group at large. In this session, we will unpack and highlight the key concepts of cultural safety. Similar to the game jam session, we will award prizes in the 4 evaluated categories. Similar to that in the intervention group, the duration of participation in the control group will be 8 hours.

### Criteria for Discontinuing or Modifying the Allocated Interventions

Participants are free to withdraw from the trial at any point. We will collect reasons for withdrawal from subjects who drop out of the trial.

Participants will not be able to switch groups once they have been randomized to the intervention or control arms, even if they request to do so. Using participants’ lists, the facilitators will ensure that participants remain in their designated groups.

### Strategies to Improve Adherence to Intervention

We will recruit 10 to 20 game jam facilitators to support participants and to ensure that all groups are able to meet the challenge of each step of the game jam protocol. The facilitators will be final-year medical students or medical interns interested in cultural safety research or game-based learning. We will train the facilitators for 1 month before the game jam to ensure that they will have the skills to support the game jam participants in their learning process successfully.

We will record attendance to the intervention and control arm activities. Along with the names of the participants, we will record the date, hour, and their signatures.

### Relevant Concomitant Care and Interventions That Are Permitted or Prohibited During the Trial

Contamination is a concern of parallel-group RCTs in education. This occurs when individuals who are receiving the intervention *leak information*, which influences results in the control group. This usually reduces the measured intervention impact, making it more difficult to find a significant difference between groups [22].

In this study, we cannot guarantee that contamination will not occur. We will minimize this risk by asking students to avoid real-time communication with their peers (eg, using their cell phones), and we will conduct intervention and control activities simultaneously in different buildings. The groups will have different lunch breaks.

### Outcomes

#### Primary Outcomes

The primary outcome is the self-reported intended patient-oriented behavior of students. This derives from the response to the statement, “I will never be open to include my patients’ cultural beliefs and practices in the health decision-making process.” We are assessing students’ *intended* behavior instead of actual practice/action. Our primary concern is sustained intention 6 months post intervention.

A supplementary analysis will examine the primary outcome in the context of a results chain using the conscious knowledge, attitudes, subjective norms, change intention, sense of agency, discussion, and behavior/action (CASCADA) model of planned behavior [23]. The model includes the following variables:

- *Conscious knowledge* was the response to the statement “I consider the cultural beliefs of my patients are not important for health decision-making.”
- *Attitude to cultural safety* was derived from the statement “It is not worth considering the cultural beliefs of my patients to improve their health.”
- *Subjective norm* used the statement “Although many physicians disapprove of cultural beliefs, I think that these beliefs could improve my patients’ health.”
- *Intention to change* was derived from the statement “I will never be open to include my patients’ cultural beliefs and practices in the health decision-making process.”
- *Agency* was the response to the statement “I feel prepared with the knowledge and skills to prudently incorporate my patients' cultural practices in the health decision-making process.”
- *Discussion* derived from the response to the statement “I will discuss cultural safety with other students and physicians so they can prudently incorporate their patients' cultural practices in the health decision-making process.”

Agency and discussion replace perceived behavior in the conventional theory of planned behavior [24]. Agency involves both self-efficacy and collective efficacy. The CASCADA model includes discussion as an additional element in the results chain toward behavior change [25]. Action as a clinician, of course, cannot be known while the student is still studying. We will extrapolate this in a supplementary analysis following the successful use of the CASCADA model to explore dengue prevention behavior [25].

#### Secondary Outcomes

Secondary outcomes comprise (1) students’ confidence (transcultural self-efficacy) in their general transcultural skills and (2) qualitative understanding of the impact of the intervention in the clinical practice of medical students and medical interns through the most significant change technique. We will assess transcultural self-efficacy at baseline, immediately following the teaching session, and 6 months post intervention, and we will conduct a qualitative assessment in both groups 6 months after the intervention.

#### Output

Each student group of the intervention arm will create a co-designed low-technology prototype of a serious game to foster cultural safety in medical students. Some of these prototypes may serve as blueprints for future fully developed games or as input for future educational videogames.

In addition to the quantitative outcomes of the RCT, we will use the qualitative most significant change narrative technique [26] to collect and analyze stories of change from the medical students 6 months after the intervention. This technique will allow us to capture meaningful changes in the students’ clinical practice, which may not be apparent from the quantitative evaluation.

### Participant Timeline

**Figure 2:**
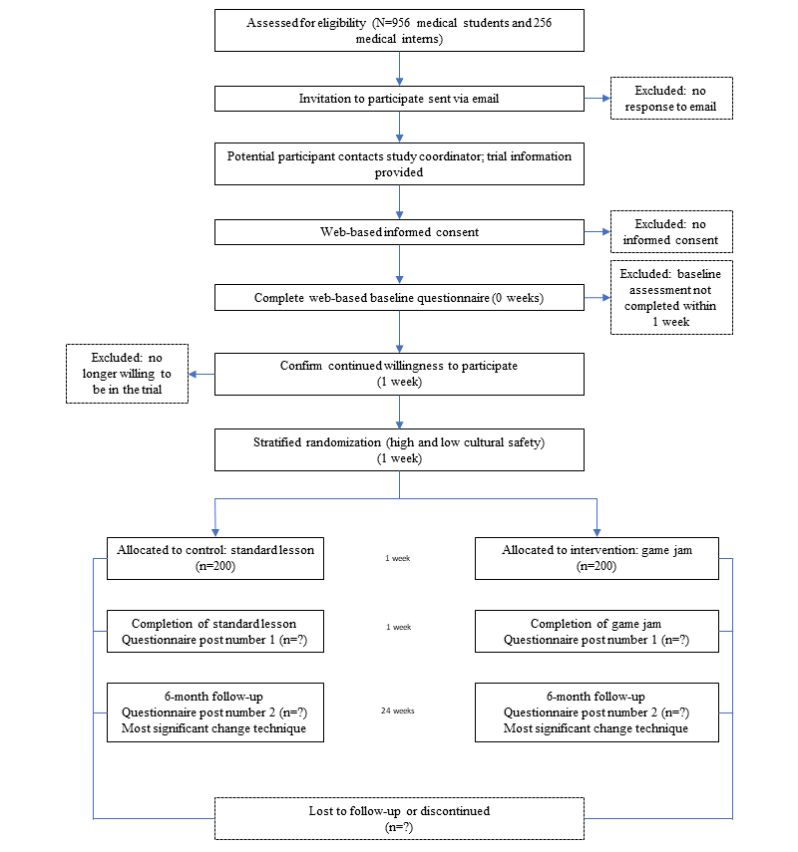
Consolidated standards of reporting trials flow diagram of the randomized controlled trial.

Figure 2 shows the consolidated standards of reporting trials flow diagram of the RCT [27].

### Sample Size

**Figure 3:**
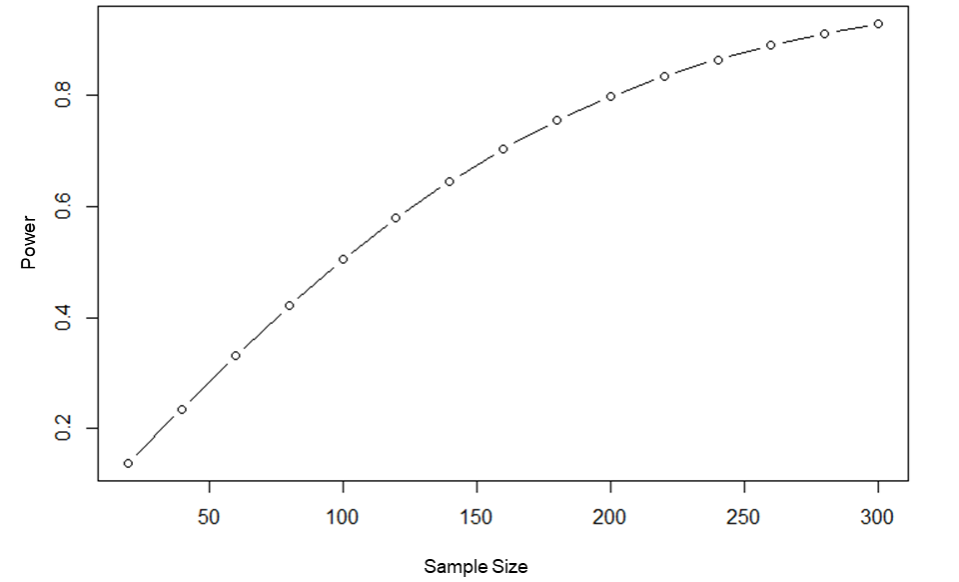
One-arm power curve for sample size calculation.

Our pilot RCT found an effect size (Cohen *d*) of 0.25 between the intervention and control arms after the teaching session (mean in the game jam group 26.9, SD 4.0; mean in the control group 25.9, SD 4.0). Using the *pwr* package in R [28], a group size of 199 participants in the game jam group and 199 participants in the control group (sample size=398) will allow detection of an effect size of 0.25, with a 2-sided α of .05 and a power of 0.8 (Figure 3). As we observed considerable contamination in the pilot RCT, 0.25 is a conservative estimate of effect size.

### Recruitment

We will contact the medical students and medical interns using La Sabana University’s mailing lists and email invitations for voluntary participation in the project. For those willing to participate, we will send further information about the project and the date and place of the intervention. We will ask interested students to complete the web-based informed consent and baseline questionnaire 1 week before the RCT.

### Allocation

A potential source of bias in our study is a possible imbalance in the level of cultural safety training between the intervention and control groups before the intervention. The reason for this issue is that in Colombia, around 40% of the population uses cultural and traditional practices to maintain their health [29]. Therefore, some students will be familiar with traditional health practices, probably making them more likely to embrace the cultural safety approach compared with students not familiar with these practices.

To address this potential bias, we will use stratified randomization based on the cultural safety score at baseline. On the basis of the preliminary results of the baseline survey, we will split the group of medical students into 2 groups: low and high level of cultural safety knowledge. Computerized randomization will allocate the students either to the intervention or control arm, and we will use equal allocation between treatment arms. The study coordinator will be responsible for generating the allocation sequence, enrolling participants, and assigning participants to interventions.

### Data Collection Methods

#### Data Collection

We will collect quantitative data at 3 time points: baseline, immediately after the intervention, and 6 months after the intervention, ‘and will collect the narratives of change only 6 months after the intervention. Participants will enter quantitative data using mobile devices and SurveyMonkey self-administered questionnaires. Similarly, they will upload their stories of change using a predesigned format on Google Forms. We report our web-based instruments in accordance with the checklist for reporting results of internet e-surveys [30] (Multimedia Appendix 2).

#### Instrument and Quantitative Data to Be Collected

To the best of our knowledge, there are no validated research instruments to measure cultural safety outcomes in health care providers. A recent systematic review [31] exploring instruments to assess cultural competence (and aligned concepts) identified 10 instruments. All of them were self-administered and based on respondent perceptions. Half of these instruments (5/10) measured cultural competence; none of them were designed to measure cultural safety.

Our recently published scoping review identified that the transcultural self-efficacy tool—multidisciplinary healthcare provider version (TSET-MHP) has been used to assess the effectiveness of game-based learning interventions to promote cultural competence [32]. Researchers report a growing body of evidence supporting the validity and reliability of the instrument [33]. The instrument assesses cognitive, practical, and affective learning dimensions that can be categorized within the classic knowledge, attitudes, and skills behavior change outcomes.

Brascoupé points out that cultural competence provides a foundation for cultural safety [34]. Ramsden sees cultural safety training as a dynamic process moving from cultural awareness to cultural sensitivity to cultural safety [3]. Following this rationale, we will use a 30-item instrument comprising 3 parts. The first part (5 items) will explore the sociodemographic characteristics of the students. These includes sex, age, level of training, place of birth, socioeconomic status, and traditional health practices used in the family. The second part (15 items) will be based on the Likert-type TSET-MHP and will explore transcultural self-efficacy.

For the third part of the instrument (cultural safety), we developed a Likert-type preliminary version based on our CASCADA variables (*Primary Outcomes* section) and tested it for validity and reliability in our pilot RCT.

#### Validity and Reliability

Using data from our pilot RCT, we followed the process proposed by Jeffreys [35] to improve the validity and reliability of the third part of our instrument. In the pilot study, the questionnaire included the following open question: *How can we improve this instrument?* An inductive thematic analysis [36] of responses identified suggestions to adjust our survey. We shared the adjusted version of the instrument by email with 2 general practitioners, 1 medical intern, 6 medical students, and 4 cultural safety experts. We adjusted the instrument according to their comments and agreed on the content validity of the instrument by consensus.

To increase the construct validity of our instrument, we used the contrasted group approach, which explores the difference between 2 separate groups [35]. To increase the predictive validity of our instrument, we looked at the score difference between 2 time points [37]. Reliability explores the degree of accuracy and consistency in measurement. Using R Studio v1.1.419, we calculated Cronbach α [38] to determine the internal consistency of our instrument. As our instrument was short (<10 items), we expected a value of >0.5 [39]. We complemented the reliability exploration using the test-retest method to explore the stability of the instrument [35]. We report the validity and reliability results of our instrument in the pilot RCT, which is not yet published.

#### Qualitative Data to Be Collected

To explore students’ stories of change after cultural safety training, we will use the most significant change approach, which is a narrative technique that allows participants to communicate changes that are most meaningful to them [40]. Using a predefined format in Google Forms, we will ask participants to write down and enter their stories based on the following instruction: “Please, tell me a story describing what you think is the most significant change in your clinical practice as a result of your participation in the activity [game jam or standard lesson] 6 months ago.”

The instructions will clarify that participants should feel free to write down stories of negative changes or to say that they did not experience any change at all. Only medical students involved in clinical practice and medical interns (third to seventh year of medical school) will be invited to participate in this part of the RCT.

#### Methods to Maximize Completeness and Quality of Data

The study coordinator and facilitators will be physically present while collecting the data at each time point to ensure the completeness of data. In addition, we will use several validation options to increase the quality of the data: specific number range, specific character range, date validation, email address format, and prompts that alert participants when they enter incomplete or invalid answers.

In this study, the familiarity of millennial and generation Z medical students with technology and computer-based education supports using web-based questionnaires should decrease social desirability bias [41]. Assured of anonymity, respondents should be less concerned about what others may think about their responses, including peers and professors [42]. Data reliability in web-based questionnaires is reportedly equal to or better than that in traditional paper-based approaches [43]. Examples include data on self-reported perceived health status, oral contraceptive use, and smoking and alcohol use. Web-based questionnaires are also faster to complete and are typically cheaper than traditional approaches, making them ideal for our research.

#### Methods for Ensuring Secure Data

SurveyMonkey and Google Forms responses are stored in a worksheet that can only be accessed through an account log-in. Data transmission uses the secure sockets layer to encrypt information during transport. After downloading the data, we will delete it from the SurveyMonkey and Google Forms. We will store the data securely for 7 years and then destroy them in accordance with Centro de Investigación de Enfermedades Tropicales (Tropical Disease Research Centre) guidelines for security, storage, and eventual destruction of data records [44].

### Methods for Analyzing Data

#### Primary Analysis

Using an intention-to-treat approach, we will perform a *t* test with 95% CIs to determine the effect of the intervention on change intention between parallel intervention and control groups 6 months after the intervention. We will assess the influence of this primary outcome in the results chain using the CASCADA approach developed by Andersson et al [25]. Transitive closure estimates the net influence of each element of the results chain on each other and on the final outcome—behavior change in practice [45].

#### Secondary Analysis

We will examine the residual impact of key baseline and sociodemographic baseline characteristics, including clustering (workgroup during the intervention or control activities), on the primary outcome. We will examine the residuals for the model assumptions and goodness of fit. This will rely on the Mantel-Haenszel approach adjusted for cluster and unconditional linear regression.

#### Supplementary Analysis

We will explore other parameters of impact, including within-group comparisons (baseline and postintervention 1 and 2) and between-group comparisons (treated versus control immediately postintervention). We will consider possible interactions with previous cultural safety training, family use of traditional medicines, and social class of participants. Planned subgroup analyses include gender, age, and social class, also using generalized linear mixed modeling with cluster as a random effect. All statistical tests will be 2-sided at a .05 level of significance. The Bonferroni method will adjust the level of significance for testing for secondary outcomes to maintain the overall level at α .05. We will express results as odds ratio/relative risk reduction for binary outcomes, standard errors, corresponding 2-sided 95% CIs, and associated *P* values.

#### Missing Data

There is no reason to expect differential missing data between game jam and standard lesson groups. We will document missingness and analyze missing data using Amelia II [46] to impute values for missing data with an expectation-maximization algorithm for the primary outcome. Estimates will reconcile data from 10 imputed datasets using Rubin’s approach [47] in the R package Zelig [48]. In addition, we will provide an attrition diagram (eg, the proportion of participants completing the surveys in each group plotted over time) [49] demonstrating the engagement of participants over time.

#### Nonstatistical Methods

Students will enter their narratives of change on the web. Using ATLAS.ti 8, 2 research assistants will individually analyze the transcripts following a deductive thematic analysis approach. In a deductive analysis, a theory aligned with the researchers’ interest drives the data analysis [36]; we will use the steps described by the CASCADA model to identify themes of change in the stories.

### Ethics

This RCT applies the ethical principles in the tri-council policy statement [50] and was approved by the institutional review board of the Faculty of Medicine at McGill University (approval number A05-B37-17B) and by the Subcommittee for Research of the Faculty of Medicine at La Sabana University (approval number 445). We will explain the confidentiality and anonymity mechanisms and the voluntary nature of participation and obtain informed consent from participants before the study.

The facilitators will ensure that each participant has signed a web-based informed consent form before proceeding with any research activity. They will be available to explain the purpose of the study, potential risks and benefits, the confidentiality of responses, and the respondents’ rights to not answer certain questions or to end their participation in the study.

## Results

Study enrollment began in July 2019. A total of 531 students completed the baseline survey and were randomized. Data collection is expected to be complete by July 2020, and results are expected in October 2020. The study was approved by the institutional review board of the Faculty of Medicine at McGill University (May 31, 2017).

## Discussion

This will be the first medical education RCT using a game jam as an educational intervention. The focus of game jams to date has been on their products, which are generally video games. Our proposal is to explore the transformative engagement occurring as a result of participating in a game jam.

Answering our research question will advance the current knowledge on game jam research and participatory design in game learning. More importantly, implementing this project will contribute to the exploration of new strategies to solve the challenges of cultural safety training in medical education, taking into consideration the time pressure in medical studies and the expectations and needs of millennial medical students.

Some have recently advocated for the need to promote cultural safety rather than cultural competence [51]. To the best of our knowledge, this will be the first initiative using the cultural safety approach in South America. Similarly, cultural safety has been traditionally restricted to the indigenous context [34], and this will be one of the first experiences to apply cultural safety in a non-Indigenous setting.

Benefits from this project include medical students gaining broader tools for their future work, including openness and dialogue about cultural and traditional health practices. This aspect will be especially relevant for them as most Colombian medical students must work for at least 1 year in a rural area as part of their compulsory 1-year return service.

Long-term potential benefits derived from the project include enhanced quality in Colombian health services, improved reputation of health institutions (higher patient satisfaction, better physician-patient relationship, and better patient adherence), and reduced health disparities among culturally diverse patients in Colombia. Assessing these outcomes is, however, outside the scope of our study.

### Challenges

We recognize several challenges. The participatory design of serious games is an emerging field, and evidence of its impact is scarce [52]. There are no agreed methodological frameworks or consensus on operational definitions. This could lead to unexpected challenges, hindering the research process. To address this issue, we conducted a pilot RCT with 79 final-year medical students to explore the acceptability and feasibility of cultural safety training through co-designed game learning, master the skills required to conduct a full-scale co-designed game learning session, pilot research methods and procedures, explore the validity and reliability of our research instrument, and identify logistical problems that might hinder the full-scale study. This helped us to understand and solve, in advance, some of the challenges. We will publish the results of the pilot RCT soon.

It is likely that only students interested in cultural safety, game learning, or research will agree to participate in the study. We will implement measures suggested by Kahan et al [53] to prevent self-selection bias in our study. We will use computerized randomization, and all students will have equal probability to be randomized to the intervention or control arm. Although blinding is nearly impossible in RCTs applied to education research, the students will not be aware of the allocation sequence or what group they were allocated to. They will only have knowledge about the auditorium that each of them should attend on the day of the intervention. Our facilitators will prevent students from deliberately switching their allocation status. Finally, 5 facilitators in each study arm site will ensure that participants remain in their designated groups (game jam or standard lecture).

Some argue that the reproducibility of educational interventions is hard to ensure because of the *specific teacher effect* where the results of an intervention stem from the skills of a particular teacher [54]. To maximize the reproducibility and generalizability of our intervention, we will follow the recommendations provided by the *British Medical Journal* [55]. This involves describing the intervention rigorously enough to allow its reproducibility and scrutiny in the future. We will report details about the teachers (eg, background, years of experience, and fields of expertise) and the teaching interventions (duration, education content, and pedagogical approach).

In this project, we will assess education-related outcomes based on the theory of planned behavior. Experts in cultural safety training recommend, however, the use of patient-related outcomes such as evaluations of care, health outcomes, involvement in care, and health behaviors to assess cultural safety interventions [56]. Assessing patient-related outcomes would require a more complex approach that goes beyond our logistical and economic capacity. The impact assessment, however, will include a qualitative understanding through the most significant change evaluation. This will document the narratives of change in the clinical practice of medical students.

The findings of this project will be specific to the Colombian cultural context. In Colombia, exploring ethnocentrism and cultural safety is simplified by the widespread use of traditional health practices [29]. In other settings, where cultural and traditional health practices are not widespread, this approach will be less relevant, and it might be necessary to confront ethnocentrism in a more abstract way or through other stigmatizations.

## Appendix

Multimedia Appendix 1 Standard protocol items: recommendations for interventional trials checklist of the study protocol.

Multimedia Appendix 2 Checklist for reporting results of internet e-surveys.

## Abbreviations

CASCADA: conscious knowledge, attitudes, subjective norms, change intention, sense of agency, discussion, and behavior action
RCT: randomized controlled trial
TSET-MHP: transcultural self-efficacy tool—multidisciplinary healthcare provider version
