# Supplementary material for: Impact of Co-Designed Game Learning on Cultural Safety in Colombian Medical Education: Protocol for a Randomized Controlled Trial
Source: JMIR Res Protoc. 2020 Aug 31;9(8):e17297. doi: 10.2196/17297 (PMC7490681; doi:10.2196/17297)
Supplement: Multimedia Appendix 2 [file resprot_v9i8e17297_app2.docx]

**Multimedia Appendix 2. Data reporting guidelines, checklist for reporting results of internet E-Surveys (CHERRIES)**

| ***Item category*** | ***Checklist item*** | ***This study*** |
| --- | --- | --- |
| **Design** | Describe survey design | Target population: Undergraduate medical students and medical interns at *La Sabana* University in Colombia.  Purposive sample. We will contact the medical students and medical interns using *La Sabana* University’s mailing lists and will e-mail invitations for voluntary participation in the project. For those willing to participate, we will send further information about the project as well as the date and place of the intervention. We will ask interested students to complete the online informed consent and baseline questionnaire one week before the RCT. |
| **IRB approval and informed consent process** | IRB approval | This RCT was approved by Institutional Review Board of the McGill’s Faculty of Medicine (approval number A05-B37-17B) and by the Sub-committee for Research of the Faculty of Medicine at La Sabana University (approval number 445). |
|  | Informed consent | Informed consent was provided by completing the e-survey. |
|  | Data protection | SurveyMonkey and Google responses are stored in a worksheet that can only be accessed through an account login. Data transmission uses Secure Sockets Layer to encrypt information during transport. The data storage is as secure as most other systems that store survey information. After downloading the data, we will delete it from SurveyMonkey and Google Forms. We will be responsible for ensuring the data are securely stored for seven years and then destroyed in accordance with CIET guidelines for security, storage, and eventual destruction of data records. |
| **Development and pre-testing** | Development and testing | We will use a 30-item instrument comprised of three parts. The first part (five items) will explore sociodemographic factors of the students. The second part (15 items) will be based on the Transcultural Self–Efficacy Tool and will explore knowledge, attitudes, skills, and behaviors of cultural competence. For the third part of the instrument (cultural safety), we developed a Likert-type preliminary version based on our CASCADA variables (see primary outcome) and piloted it for validity and reliability in our pilot RCT.  Regarding the qualitative data, using a pre-defined format in Google forms, we will ask participants to write down and enter their stories. |
| **Recruitment process and description of the sample having access to the questionnaire** | Open survey versus closed survey | Closed survey sent by institutional email from *La Sabana* University. |
|  | Contact mode | Contact with participants online through e-mail and face-to-face. |
|  | Advertising the survey | Not applicable. |
| **Survey administration** | Web/E-mail | E-mail survey using SurveyMonkey and Google Forms. There was an automatic method for capturing responses. |
|  | Context | We will contact the medical students and medical interns using *La Sabana* University’s mailing lists and will e-mail invitations for voluntary participation in the project. |
|  | Mandatory/voluntary | Voluntary survey. |
|  | Incentives | Participants of the qualitative component of the study will enter a raffle after submitting their story. We will offer one gift card of CAD 20 for every ten stories collected. |
|  | Time/date | July 2019 to July 2020. |
|  | Randomization of items or questionnaires | Stratified randomization by cultural safety score at baseline. |
|  | Adaptive questioning | Not applicable. |
|  | Number of items | 30 items in total. |
|  | Number of screens (pages) | Three Webpage per survey. |
|  | Completeness check | We will use several validation options to increase the quality of the data: specific number range, specific character range, date validation, email address format, and prompts that alert participants when they enter incomplete or invalid answers. |
|  | Review step | Respondents were not able to review and change their answers. |
| **Response rates** | Unique site visitor | Repeat visitors will not be excluded. Some students will share their devices with students who do not have access to an electronic device. |
|  | View rate (ratio of unique survey visitors/unique site visitors) | Not available. |
|  | Participation rate (ratio of  unique visitors who agreed to participate/unique first  survey page visitors) | Not available. |
|  | Completion rate (ratio of users who finished the survey/users who agreed to participate) | Not available. |
| **Preventing multiple entries from the same individual** | Cookies used | Repeat visitors will not be excluded. |
|  | IP check | Repeat visitors will not be excluded. |
|  | Log file analysis | Repeat visitors will not be excluded. |
|  | Log file analysis | Repeat visitors will not be excluded. |
|  | Registration | Repeat visitors will not be excluded. |
| **Analysis** | Handling of incomplete questionnaires | Only completed questionnaires will be analyzed. |
|  | Questionnaires submitted with an atypical timestamp | Not applicable |
|  | Statistical correction | Not applicable |
